# Clinical, Diagnostic and Prognostic Characteristics of Primary Cutaneous Gamma Delta T-cell Lymphomas

**DOI:** 10.1007/s44228-022-00011-9

**Published:** 2022-06-22

**Authors:** Ibrahim N. Muhsen, Riad El Fakih, Mehdi Hamadani, Hillard M. Lazarus, Mohamed A. Kharfan-Dabaja, Mahmoud Aljurf

**Affiliations:** 1 Department of Medicine, Houston Methodist Hospital, Houston, TX USA; 2 Oncology Center, King Faisal Specialist Hospital and Research Center, PO Box 3354, Riyadh, 11211 Saudi Arabia; 3 BMT and Cellular Therapy Program, Department of Medicine, Medical College of Wisconsin, Milwaukee, WI USA; 4 Division of Hematology and Oncology, Case Western Reserve University, Cleveland, OH USA; 5 Division of Hematology-Oncology and Blood and Marrow Transplantation and Cellular Therapies Program, Mayo Clinic, Jacksonville, FL USA

**Keywords:** Gamma-delta, T-cell lymphoma, Cutaneous lymphomas

## Abstract

Primary cutaneous *γδ* T-cell lymphoma (PCGDTL) is a rare subtype of non-Hodgkin lymphoma (NHL) that arises from T-cells with *γδ* T-cell receptors. The exact incidence of PCGDTL is unknown, as it is usually lumped with other cutaneous lymphomas, which are also uncommon. It is one of the peripheral T-cell lymphoma (PTCL) subtypes which is known to have a dismal prognosis due to poor response and the paucity of available therapies. Despite the rarity and uncertainties of PCGDTL, a number of studies over the past decade were published about the pathologic, diagnostic, cytogenetic and clinical features of this disease. These diagnostic advances will open the doors to explore new therapeutics for this rare entity, specifically targeted and immune therapies. In this review, we highlight these advances, summarize the contemporary treatment approaches, and shed the light on future potential therapeutic targets.

## Introduction

T-cells are classified into *ɑβ* or *γδ* T-cells based on their T-receptor subtype. The *γδ* constitute less than 5% of circulating T-cells, but are abundant in specific tissues, particularly the spleen’s red pulp [1–3]. T-cell lymphomas are less common than B-cell lymphomas, and originate mainly from *ɑβ* T-cells, with far fewer cases originating from *γδ* T-cells [4]. *γδ* T-cell lymphomas are classified by the 2016 World Health Organization (WHO) into two main categories: primary cutaneous *γδ* T-cell lymphomas (PCGDTL) and hepatosplenic T-cell lymphoma (HSTL) [5, 6]. The prognosis of these lymphomas is generally worse than that of the *ɑβ*-T cell lymphoma counterpart. Given the rarity of both PCGDTL and HSTL, there is a paucity of literature on the clinical features of these entities, and their available treatment options [4, 7].

PCGDTL is a type of primary cutaneous lymphoma (PCL). PCLs are non-Hodgkin lymphomas limited to skin, with no extra-cutaneous manifestations at diagnosis [8, 9]. They are generally uncommon and relatively newly recognized and classified [10]. The WHO classification in 2016 [6] and the revised WHO- European Organization for Research and Treatment of Cancer (EORTC) classification of PCL in 2018 [11] categorize PCL into T-cell and B-cell lymphomas with multiple subtypes under each.

Primary cutaneous T-cell Lymphoma (PCTCL) is more common than cutaneous B-cell lymphoma, with Mycosis Fungoides (MF) being the most common subtype. The majority of PCTCL are derived from *ɑβ* T-cells. Conversely, PCGDTL is generally derived from *γδ* T-cells. This review aims to provide an update on the current understanding of the clinical, diagnostic, and treatment aspects of PCGDTL.

## T-Cell Receptor and the Gamma-Delta Subtype

The T-cell recognition of antigens takes place via T-cell receptors (TCR). The TCR consists of the antigen-binding site, which is a heterodimer of *ɑβ* (more common) or *γδ* (less common) chains [3, 12]. These chains are linked with CD3 subunits, which are critical in allowing TCR insertion into the cell membrane and, later, to mediate the signal through the TCR. The mechanism of assembly of the TCR-CD3 complex is poorly understood [13]. Genes for TCR-*ɑ* and TCR-*γ* are located on chromosome 14, whereas the genes for TCR-*β* and TCR-*δ* are on chromosome 7 [14]. Due to the development of *γ* and *δ* before *ɑ* and *β* chains, and the deletion of *δ* genes secondary to *ɑ* gene rearrangement, the formation of *ɑβ*-TCR or *γδ*-TCR is mutually exclusive [3, 12].

TCR diversity is mainly the product of the rearrangement in the Variable (V), Joining (J), Diversity (D), and Constant (C) segments [15]. However, additional factors contributing to TCR diversity include the diversification by adding nucleotides to the rearranged genes, and the multiple possibilities of pairing of different *ɑβ* or *γδ* chains [15, 16]. Hence, the ability of TCR to identify limitless antigens.

The thymus plays a crucial role in T-cell maturation by ensuring their functionality and removal of non-functional or auto-reactive T-cells. However, it has been reported that *γδ*- T cells are less dependent on thymic maturation [17]. After maturation, *γδ*- T cells are usually distributed to different organs (spleen, lymph nodes, skin, and peripheral blood), and can be usually classified as intra-epithelial or lymphoid-tissue associated [4]. Subsets of *γδ*- T cells are classified based on their corresponding variable region of the *δ* chain. The primary two subsets are V1*δ* and V2*δ*, constituting more than 97% of *γδ*- T cells in healthy individuals. Each of these subsets is predominantly found in specific tissues (V1*δ* in thymus and spleen, and V2*δ* in peripheral blood and lymph nodes) [3, 18, 19].

The immunologic role of *γδ*- T cells is not fully understood. Their presence in higher concentration on mucosal surfaces indicates a possible role in the initial phase of infection, with features of innate and adaptive immunity [20, 21]. *γδ*- T cells can recognize antigens that *ɑβ* T-cells are not able to, including some non-processed MHC proteins, lipid antigens, and direct bacterial antigens [22]. Additionally, studies have highlighted the similarities of the *γδ* T-cells to NK-cells when activated, including cytotoxic release and CD expression, indicating possible similarities in the roles of the two cell types [23]. Moreover, the reported expansion of *γδ*- T-cells in certain autoimmune diseases and cancers indicate other possible roles of these cells [24, 25].

## Epidemiology and Clinical Presentation

According to the 2018 update of the WHO-EORTC classification, the incidence of PCGDTL is less than 1% of PCLs [11]. Similarly, the incidence of PCGDTL was 1.2% among 1553 cases reported to the “T-cell project” registry [26]. Being such a rare entity, it is not unexpected that large scale epidemiological studies are lacking and, as such, the pathogenesis and prognosis of PCGDTL remain poorly understood. In a case series involving 53 patients, 23% had a history of autoimmunity, with around 10% having lymphoproliferative disorders and/or carcinomas [27]. Similar associations have been reported in a smaller case series [28]. This might be explained by the tissue-restricted immunologic stimulation leading to polyclonal proliferation and transformation [3].

Table 1 is a summary of the data reported in case series and retrospective studies of more than ten patients. In the majority of reported cases, the patients’ age was typically above 50 years, with a median ranging from 50 to 60 years, and no suggestion of gender-distribution difference [26, 27]. Clinically, deep plaques are the most frequent presentation [27–29]; however, nodules, patches and superficial plaques can also be the presenting feature. Ulceration was reported in up to 50% of cases [27]. In most studies, patients presented with multifocal lesions, except in one report where all patients presented with localized/ solitary lesions [30]. Panniculitis-like presentation has also been reported [31]. Additionally, many patients present with associated B-symptoms [26, 29, 32, 33].

**Table 1:** PCGDTL patients’ characteristics as reported in multiple case series & retrospective studies

	Daniels et al. [29]	Foss et al. [26]	Jour et al. [30]	Toro et al. [31]	Willemze et al. [11]	Guitart et al. [27]	Gibson et al. [32]	David et al. [47]
*General patient’s characteristics*								
Sample size (*n*)	25	19	10	33	20	53	10	48
Median age (range)	–	–	51 (2–89)	49 (13–82)	59 (13–79)	61 (25–91)	42 (26–63)	62 (20–88)
Female (%)	–	–	70	42	65	44	–	33
Median follow-up (months)	7.5 (0–77)	77	–	–	–	18 (4–108)	49 (3–104)	–
B-Symptoms (%)	43	44	–	–	65	NR	60	40
Localized *N* (%)	–	–	100	–	25	25	–	–
Multifocal *N* (%)	–	–	0	–	75	75	–	–
*Immunophenotypical features*								
CD2 (%)	64	–	–	–	–	–	84	–
CD7 (%)	12.5	–	89	–	–	24	40	–
CD56 (%)	69	–	83	–	60	38	–	–
CD5 (%)	21	–	–	–	–	12	57	–
CD4 (%)	5	–	0	–	5	7	12.5	–
CD8 (%)	18	–	33	–	10	40	37.5	–
Granzyme B (%)	70	–	–	–	100	76	100	–
TIA (%)	74	–	–	–	100	92	100	–

## Laboratory Findings

### Serologic and Radiologic Findings

There are no specific laboratory findings in PCGDTL. However, two commonly reported findings in the literature are lactate dehydrogenase (LDH) elevation and the presence of cytopenias. The finding of the former is variable: some series reported no LDH elevations, while, in others, up to 30% of cases had an elevated LDH [26, 27, 32]. This variability is likely related to differences in disease burden. Cytopenias, on the other hand, were noted in the literature in isolation or associated with other laboratory abnormalities as a part of hemophagocytic lymphohistiocytosis (HLH). HLH was frequently reported in patients with PCGDTL, with variability in prevalence ranging from 0 to about 50% in some studies [31–33]. For instance, Willemze, et al. found HLH in 9 out 20 cases (45%). The presence of HLH on presentation adversely impacted the outcome of these patients, as HLH was fatal in seven of the nine patients. This has also been reported more frequently with the subcutaneous subtype of PCGDTL [34]. Imaging in the form of a CT scan or a PET scan are important components of PCGDTL staging. The role of imaging is mainly to stage PCGDTL, and to rule out extra-cutaneous involvement. A staging approach using the tumor (T), nodes (N), and metastases (M) classification (TNM) of cutaneous lymphomas other than MF and Sezary syndrome (SS) was proposed by the International Society for Cutaneous Lymphomas (ISCL) and the Cutaneous Lymphoma Task Force of the EORTC [35]. The approach is based on the size and location of lesions, nodal involvement, and extracutaneous non-lymph node disease.

### Histopathological Features and Detection of Gamma-Delta Expression

Three major histological patterns have been reported based on the skin layer involved, including the epidermal, dermal, and subcutaneous (panniculitis) patterns. The cases with predominant dermal or subcutaneous components are thought to be less common [28].

Multiple epidermal histological features were described in the literature, including spongiosis, parakeratosis, and acanthosis [28]. Merrill et al. [28] found that lymphomas with mainly epidermal involvement are more likely to have an epidermotropic pattern than those with dermal or subcutaneous predominant components where the diffuse histologic pattern was frequently seen. Interface changes were more common in the predominantly epidermal lymphomas. Multiple cases with the latter were reported to have an indolent course, compared to cases with predominantly subcutaneous and dermal involvement [36]. However, other reports showed no difference between the two histologic presentations [37].

Previously, the absence of *ɑβ*- TCRs was an indirect way to identify *γδ* T-cells. Currently, *γδ* T-cells are identified by the presence of *δ*-1 TCR chain in frozen tissues and formalin-fixed paraffin-embedded (FFPE) tissues [38]. A monoclonal antibody to the TCR *γ*-chain was also used in recent years, but it is not consistently available [39]. Other potential reagents are under evaluation [39].

### Immunophenotypical Features

The immunophenotypical features reported from different case series are summarized in Table 1. CD3 is expressed in almost all the cases, but is not specific and does not help differentiating PCGDTL from other T-cell lymphomas. Other frequently identified markers include CD2, CD4, CD5, CD 7, CD8, and CD56. Markers such as CD2, CD56 are more frequently expressed as compared to the others. For instance, CD2 was reported in around 60–80% of cases in certain studies, and CD56 in 40–80% of cases (Table 1). CD4 is less frequently expressed compared to CD8. Moreover, cytotoxic protein expression such as granzyme B and TIA is frequently seen, with the latter being expressed in over 90% of cases. Immunophenotypic changes and shifts have been observed in this disease, suggesting antigenic modulation [40]. CD30 expression was reported in some cases of PCGDTL [28], and this constitutes a diagnostic challenge, as it becomes difficult to differentiate PCGDTL from entities such as Lymphomatoid papulosis (LyP). However, it may help by allowing the use of targeted anti-CD30 antibodies during the therapeutic course. Other markers, such as CXCR4, have been found, and can be useful for diagnostic and therapeutic values [30].

### Cellular Basis and Genetic Mutations

Understanding the genetic makeup of PCGDTL may help identifying the altered pathways in this type of lymphoma and, potentially, target them. As discussed earlier, two subpopulations (V*δ*1 and V*δ*2) make up the majority of *γδ*- T cells in blood and tissues. V*δ*2 T-cells are more prevalent in the skin; however, the epidermal and dermal predominant PCGDTL derive mainly from V*δ*1 T-cells [29]. Isochromosome 7 alterations, chromosome 5 and 8 trisomies are reported in HSTL [41–43]. In PCGDTL, a number of cytogenetic abnormalities including amplifications, deletions, and breakpoints in different chromosomes (1q, 15q, 7 q, 9p, 14 q, and 18q) were found [29, 44]. Using targeted sequencing, Küçük et al. showed that 13% of patients (2 out of 15) had *STAT3*, and 27% (4 out 15) had *STAT5B* mutation [45]. Similarly, Daniels et al. reported their work on 25 patients with PCGDTL, showing STAT3 and STAT5B alterations [29]. Multiple other alterations affecting a number of oncogenic pathways, including MYC, JAK/STAT, MAPK, and chromatin mutations have been described [46].

## Treatment Options

### Chemotherapy and Other Targeted Agents

Currently, the most commonly cited front-line therapy approach in PCGDTL is similar to that of peripheral T-cell lymphomas. The reported frontline therapies are anthracycline based with either cyclophosphamide, doxorubicin hydrochloride, vincristine, and prednisone (CHOP) or etoposide + CHOP (EPOCH). Other combination included ifosfamide, carboplatin, and etoposide (ICE), and hyperfractionated cyclophosphamide, vincristine, doxorubicin, and dexamethasone (Hyper-CVAD). Most of these studies are in the form of case series with small number of patients, which limit the ability to draw solid conclusions. Table 2 is a summary of reported frontline therapies and outcomes of PCGDTL.

**Table 2:** A summary of frontline therapies and outcomes of PCGDTL

Report	Sample size	Regimen (% of patients)	Outcomes
Foss et al. [26]	19	Anthracycline-based chemotherapy (100%)	25% reached CR/CRu Median survival 47 months 3-year OS 72%
Willemze et al. [11]	20	CHOP or CHOP-like (70%)	30% achieved CR 35% with PD or no response 5-year OS 11%
Toro et al. [31]	23	CHOP or CHOP-like (43%)	5-year OS 15%
David et al. [47]	48	Anthracycline-based (CHOP, EPOCH, CHOEP) (11%)	19% Complete Remission after front line therapy 10% Stable disease 35% Progressive disease 15% Unknown 2-year PFS 39% 2-year OS 36%
		Bexartoene (10%)	
		Other therapies used include Brentuximab Vedotin, ICE, ESHAP	

*CHOEP* cyclophosphamide, hydroxydaunorubicin, vincristine, etoposide, prednisone; *CHOP* cyclophosphamide, hydroxydaunorubicin, vincristine, prednisone; *CR* complete respone; *CRu* complete response unconfirmed; *EPOCH* etoposide, prednisone, vicristine, cyclophosphamide, hydroxydaunorubicin; *ESHAP* etoposide, methylprednisolone, cytarabine, cisplatin; *ICE* ifosfamide, carboplatin, etoposide; *OS* overall survival; *PD* progressive disease; *PFS* progression free survival

Included review of studies prior to 2000

Data from multiple studies illustrate the suboptimal outcomes in patients with PCGDTL. For instance, the number of patients reaching complete remission was reported to be between 20 and 30% [11, 26, 47]. Moreover, the survival was variable between different studies. In the work by Foss et al. the 3-years overall survival was 72%, whereas, the 2-years overall survival was only 36% in another report [47]. Over the last two decades, the use of targeted and biomarker-driven therapies in peripheral T-cell lymphoma increased [48, 49]. Several agents targeting cell surface receptors have been used, including brentuximab, a CD30-directed therapy used in patients with Hodgkin lymphoma and systemic anaplastic large cell lymphoma [50]. Brentuximab was investigated with some efficacy in cases of CD30-positive PCDGTL [51, 52]. Mogamulizumab, another antibody that targets CXCR4, was studied in a phase III clinical trial in patients with relapsed/refractory cutaneous T-cell lymphoma (all patients had either MF or SS). In that trial, mogamulizumab, in comparison to vorinostat, showed a better progression free survival [53]. Alemtuzumab, an anti-CD52 antibody, has shown good activity in HSTL [4, 54, 55]. One case report using alemtuzumab in relapsed/refractory PCGDTL failed to show a response [56].

In addition to cell surface receptors, targeting T-cell pathways was another important therapeutic intervention that was explored in recent years. Different histone deacetylase inhibitors (HDACi) were investigated in PTCL, including cutaneous T-cell lymphoma. Vorinostat was the first to obtain approval by the FDA, followed by belinostat and romidepsin. The latter is an HDACi approved for relapsed or refractory PTCL after a phase II trial of 130 patient. Only 1 of the 130 patients treated on that trial had PCGDTL [57]. The role of immune checkpoint inhibitors including mainly PD-1, and PD-L1 inhibitors including pembrolizumab and nivolumab was investigated in multiple types of peripheral T-cell lymphomas, but results were generally not encouraging [58, 59]. Other novel checkpoint inhibitors, such as the CD47 blocker TTI-621, are being investigated in peripheral T-cell lymphomas [60]. Combinations of these novel agents with different other therapies are being tested in different types of T-cell lymphomas [61–64].

Many other therapies have shown some activity based on case reports and series. For instance, Ontak (denileukin diftitox), a CD-25 (a component of the IL-2 receptor) directed cytotoxin used in recurrent cutaneous T-cell lymphoma was tried in combination with chemotherapy. Bexarotene was used, in combination or alone, in subcutaneous panniculitis-like T-cell lymphoma, with good initial response [65, 66]. Bendamustine was used in relapsed/refractory T-cell lymphomas with 28% of patients achieving CR; however, none of these patients in this series had PCGDTL [67]. In a case report of one patient, bendamustine led to sustained complete remission for more than 12 months [68].

It is important to note that most of the studies lumped PCGDTL with other T-cell lymphomas [7, 66], thus masking it difficult to draw conclusions regarding the efficacy of these therapies in PCGDTL. More recent investigations have highlighted the genomic characteristics and the molecular pathogenesis of PCGDTL. These new findings may help to investigate new approaches in treating these patients. For instance, there is an increased recognition of the role of pathways such as KRAS, and JAK/STAT pathways [29, 69, 70].

### Hematopoietic Cell Transplant and Cellular Therapies

Transplant society guidelines, including the American Society of Transplant and Cellular Therapy (ASTCT) and the European society for Blood and Marrow Transplantation (EBMT), list hematopoietic cell transplant (HCT) as an option for advanced cutaneous T-cell lymphomas [71]. Both autologous and allogeneic HCT have been used with variable success rates. Table 3 summarizes two studies that reported the outcomes of transplant in 14 patients with PCGDTL. Thirteen out of 14 received an allogeneic HCT (the majority from a matched sibling donor) and only 1 underwent autologous HCT. The most commonly used first-line therapy was anthracycline-based combination. In one of the series, the majority of patients (70%) were in complete response (CR) at the transplant time. Five out of the seven remained in CR at the time of last follow up, while the two patients who had active disease at the time of HCT died [32]. Low-intensity conditioning (pentostatin-based) with total body irradiation was the most commonly used conditioning regimen. In the other case series, two of seven patients died in the first 100 days from acute graft-versus-host disease (aGvHD). A third patient died at day 210 from hemorrhagic cystitis complications. Two patients relapsed and responded to brentuximab [51]. Thus, hematopoietic cell transplant seems to be an option in these patients, but careful patient’s selection to avoid excessive transplant-related mortality is warranted.

**Table 3:** Case series on transplant outcomes of patients with PCGDTL

	Isufi et al. [51]	Gibson et al. [32]
Sample size (n)	7	7
Median age (Range)	53 (38–40)	42 (26–63)
*First line therapy prior to HCT*		
Anthracycline based chemotherapy	6/7	6/10
Non anthracycline based chemotherapy or other therapies	1/7	4/10
Radiation	0/10	1/10
*Number of lines of therapy before transplant*		
1	6	3
2	1	1
3 or more	0	3
*Response to last treatment*		
CR	5	5
PR	2	0
PD	0	2
*Type of transplant*		
Auto	0	1
Allogeneic	7	6
*Type of transplant in allo*		
MRD	4	5–6
MUD	1	0–1
Haplo	2	0
*Conditioning therapy*		
Myeloablative	2	1
Reduced intensity	5	5

Chimeric antigen receptor (CAR) therapy is being increasingly used and investigated in both hematologic and solid malignancies. CAR therapy trials in peripheral T-cell lymphoma are still limited. Several targets have been identified and are currently being investigated, including CD4, CD5, CD7 and CD30.

Table 4 summarizes the current clinical trials utilizing CAR therapy in T-cell lymphomas. All the studies included in this report were for relapsed/refractory T-cell non-Hodgkin lymphoma, rather than PCGDTL. However, some of these targets are expressed in cases of PCGDTL (see Table 1).

**Table 4:** Current clinical trials utilizing CAR therapy in T-cell lymphoma

Target	Study	Disease status at enrollment	Phase	Indications	Status
CD 4	NCT04712864	Relapsed/Refractory	I	PTCL-NOS AITL CTCL(either MF or SS)	Not yet recruiting
CD 5	NCT03081910	Relapsed/Refractory	I	T-ALL T-LLy T-NHL: AITL, EATL, MEITL, PTCL, NOS, ALCL, Extranodal NK/T cell lymphoma, MF/SS Stage IIB or higher	Recruiting
CD7	NCT04004637	Relapsed/Refractory	I	NK/T cell lymphoma T-LLy T-ALL	Recruiting
	NCT04264078	Relapsed/Refractory	I	T-ALL T-LLy T-NHL: AITL, EATL, MEITL, PTCL, NOS, ALCL, Extranodal NK/T cell lymphoma, MF/SS Stage IIB or higher	Not yet recruiting
	NCT02742727	Relapsed/Refractory	I/II	Acute myeloid leukemia Precursor T lymphoblast leukemia/lymphoma T- prolymphocytic leukemia T-LGL T-NHL: AITL, EATL, Extranodal NK/T cell lymphoma, HSTL	Unknown
	NCT04033302	NR	I/II	T-ALL T-LLy AML NK Cell Lymphoma	Recruiting
	NCT03690011	Relapsed/Refractory	I	T-ALL T-LLy T-NHL: AITL, EATL, MEITL, PTCL, NOS, ALCL, Extranodal NK/T cell lymphoma, MF/SS Stage IIB or higher	Not yet recruiting
CD30	NCT04526834	Relapsed/Refractory	I	T-NHL: ALCL, PTCL- NOS, ENKTCL nasal type DLBCL-NOS and PMBCL	Recruiting
	NCT04008394	Relapsed/Refractory	I	ALCL, AITL, NK/T-cell lymphoma; Peripheral T-cell lymphoma (PTCL); Hodgkin lymphoma;	Recruiting
	NCT03049449	NR	I	ALCL, AITL, PTCL-NOS, DLBCL-NOS, primary mediastinal B-cell lymphoma, EATL, Extranodal NK/T cell lymphoma	Recruiting
CD30 and CCR4	NCT03602157	Relapsed/Refractory	I	cHL CTCL(either MF or SS) Lymphomatoid papulosis Cutaneous ALCL B-cell lymphoma, unclassifiable	Recruiting

*AITL* angioimmunoblastic T cell lymphoma; *ALCL* anaplastic large cell lymphoma; *ALL* acute lymphoblastic leukemia; *AML* acute myeloid leukemia; *cHL* classic Hodgkin lymphoma; *CTCL* cutaneous T cell lymphoma; *DLBCL* diffuse large B cell lymphoma; *EATL* Enteropathy-associated T-cell lymphoma; *ENKTCL* Extranodal natural killer/T-cell lymphoma; *HSTL* hepatosplenic T cell lymphoma; *MEITL* Monomorphic epitheliotropic intestinal T-cell lymphomas *MF* mycosis fungoides; *NK* natural killer; *NOS* not otherwise specified; *NR* not reported; *PMBCL* primary mediastinal B cell lymphoma; *PTCL-NOS* peripheral T cell lymphoma; *SS* sesary syndrome; *TLGL* T-cell large granular lymphocytic leukemia; *T-LLy* T lymphoblastic lymphoma; *T-NHL* T cell non hodgkin lymphoma

**Fig. 1:**
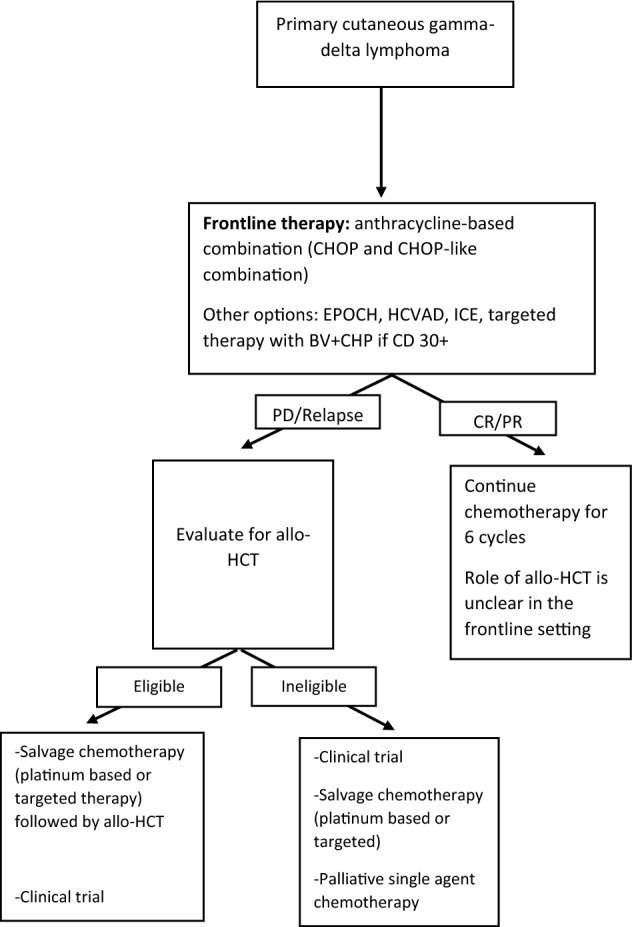
Suggested algorithm for management of primary cutaneous gamma-delta lymphoma. *Allo-HCT* allogeneic hematopoeitic cell transplant; *BV-CHP*: brentuximab vedotin, cyclophosphamide, hydroxydaunorubicin, prednisone; *CHOP* cyclophosphamide, hydroxydaunorubicin, vincristine, prednisone; *CR* complete respone; *EPOCH* etoposide, prednisone, vicristine, cyclophosphamide, hydroxydaunorubicin; *HCVAD* hyperfractionated cycyclophosphamide, vincristine, adriamycin and dexamethasone; *ICE* ifosfamide, carboplatin, etoposide; *PD* progressive disease; *PR* partial remission

Figure 1 is the authors’ suggested algorithm for management of PCGDTL.

## Prognosis

Compared to other T-cell cutaneous lymphomas, PCGDTL appears to have a worse prognosis. As shown in the different case series, the 5-years survival was less than 20% [11, 31, 72]. However, a recent publication by Foss et al. [26] reported an encouraging 3-years survival of 72%. Identifying prognostic factors is challenging, due to the small number of cases. Factors such as histopathologic findings or clinical features have been analyzed in an attempt to understand their impact on the prognosis. The histopathologic features appear to be prognostic, with better survival in patients presenting with epidermotropic involvement, rather than dermal or subcutaneous (panniculitis) involvement [28, 31].

## Research Agenda and Future Directions

Despite being recognized more than 20 years ago, our understanding of PCGDTL is still limited, in part secondary to the rarity of this disease. The following are directions that we hope that future research will accomplish:

- More studies are needed to elucidate the molecular and genetic alterations in this disease which, in turn, can help better understanding of how to use targeted therapies directed to these alterations.
- Targeted therapies, including immunotherapies already showing efficacy in a number of lymphomas, including T cell lymphomas, and are worth exploring in PCGDTL.
- A collaborative, international, multi-institutional approach is definitely needed, as the number of cases of PCGDTL are limited worldwide.
